# Association of plasma fatty acid alteration with the severity of coronary artery disease lesions in Tunisian patients

**DOI:** 10.1186/s12944-017-0538-y

**Published:** 2017-08-14

**Authors:** Samia Hadj Ahmed, Nadia Kaoubaa, Wafa Kharroubi, Amira Zarrouk, Mohamed Fadhel Najjar, Fathi Batbout, Habib Gamra, Gerard Lizard, Mohamed Hammami

**Affiliations:** 1 Faculty of Medicine, Research Laboratory LR12ES05, Lab-NAFS ‘Nutrition - Functional Food & Vascular Health’, University of Monastir (Tunisia), Avicene st, 5019 Monastir, Tunisia; 2 Biochemistry Department CHU Fattouma Bourguiba Monastir- Tunisia, Monastir, Tunisia; 3 Cardiology Department CHU Fattouma Bourguiba Monastir- Tunisia, Monastir, Tunisia; 4 Team ‘Biochemistry of Peroxisome, Inflammation and Lipid Metabolism’ EA 7270 /University of Bourgogne-Franche Comté / INSERM, Dijon, France

**Keywords:** Fatty acid profile, Coronary artery disease, Gensini score, Desaturation index

## Abstract

**Background:**

Some factors related to diet are known to be involved in the progression of atherosclerosis in humans.

**Methods:**

The relationship between plasma fatty acid (FA) levels and the severity of coronary artery disease (CAD), evaluated by Gensini score (GS), was investigated in CAD Tunisian patients compared to controls. Lipid profiles were analyzed, GS was calculated in CAD and non-CAD patients and compared to controls.

**Results:**

CAD patients showed an alteration of conventional lipid parameters. In fact, a significant increase of plasmatic triglycerides (TG) level, atherogenic lipid ratios (TC/HDL-C,TG/HDL-C, LDL-C/HDL-C); and ApoB/ApoA1 was observed in the CAD group comparatively to controls (*p* < 0.001).

Gensini score was showed to be a good indicator to evaluate cholesterol metabolism disorders associated with HDL-C since a negative association was found between HDL-C levels and GS for the two groups of patients. In addition, in the relation with FA and classes of FA, a negative association was found as expected, between Gensini score and total MUFA, PUFA n-3, total PUFA, GLA, DGLA and DHA. Furthermore, a positive association with stearic and erucic acid was found. Suggests that, GS is also a good indicator to evaluate FA metabolic disorders. Higher elongation index and modifications of desaturation index (D5D, D6D and D9D) were observed in patients compared to controls, supporting FA metabolism modifications.

**Conclusions:**

In conclusion, although that Tunisian population appears to follow the Mediterranean diet, variations of plasmatic FA levels and desaturase activities in CAD patients highlights an alteration of FA metabolism and suggests an important implication of certain FA in the development of atherosclerosis.

## Background

Coronary artery disease (CAD) is a common term for the buildup of the plaque in the heart’s arteries that could lead to heart attack. It remains the leading cause of death in different countries in the world. Altered cholesterol metabolism and pro-inflammatory mechanisms have been associated with the development of atherosclerotic plaques [1].

In addition to dyslipidemia, diabetes mellitus, hypertension, obesity, which are risk factors for the development of atherosclerosis [2], fatty acid metabolic abnormalities became also an important risk factor. The severity of CAD is evaluated by a Gensini score (GS) [3], which became the standard score reflecting the vascular severity of coronary lesions.

The relation between diet and CAD has been studied intensively for nearly a century and FA lipotoxicity has gradually became a new research point. Dietary fat plays an important role in heart disease by affecting atherogenesis, thrombosis, and coronary infarction [4].

FA are classified as saturated fatty acids (SFA), monounsaturated fatty acids (MUFA), and n-6 and n-3 polyunsaturated fatty acids (n-6 PUFA) (n-3 PUFA). All these fatty acids are known to have different effects. A high intake of SFA is now considered to be a positive risk factor for coronary heart disease (CHD) which accelerates atherogenesis, whereas MUFA and PUFA generally reduce CAD by reducing plasma lipids [5]. It was also proved that a low consumption of SFA and a proportionally higher intake of n-6 PUFAs, were associated with a significant reduction of CAD [5], and that n-3 PUFA had a preventive effect on CAD by modulating eicosanoid synthesis and thus may have potential for the amelioration of atherogenesis and thrombosis.

Several studies have reported an inverse relation between linoleic acid (LA, 18:2n-6) concentration and risk of atherosclerosis showing its role to reduce cardiovascular risk [6]. Dietary eicosapentaenoic acid (EPA, 20:5n-3) and docosahexaenoic acid (DHA, 22:6n-3) can also prevent acute coronary syndrome, and heart failure [7]. The concentration of plasma FA is influenced not only by dietary intake but also by the endogenous metabolism of FA and genetic variation [8].

In humans, there are three key enzymes in FA metabolism which are stearoyl-CoA desaturases (SCD), known as delta-9-desaturase (D9D), catalyzing the synthesis of MUFA; delta-5-desaturase (D5D) and delta- 6-desaturase (D6D) catalyzing the endogenous synthesis of long-chain unsaturated FAs. Recently D9D has been found as an independent risk factor of cardiovascular disease [9].

These enzymes play a crucial role in the mediation and modulation of metabolic functions and physical properties of the cell [10]. FA ratios used as surrogate measures of desaturase activities might be as important for metabolic changes as individual FAs which have been related to metabolic diseases and CAD [11].

Our study aims to evaluate the relation between dietary fat biomarkers, desaturase indexes, and CAD in a Tunisian population.

## Methods

### Patients

A total of 150 Tunisian patients consulting for coronary disorders were recruited from F. Bourguiba University Hospital (Monastir, Tunisia), and were clinically examined by coronary angiography at the department of Cardiology. A GS was calculated for all these patients in order to estimate the severity of CAD. According to angiographic data, the patients were divided into two subgroups: 111 Tunisian patients with CAD (80 men; 31 women) and 39 Tunisian patients without CAD but with potential cardiovascular risk factors (16 men, 23 women).

Patients with CAD, which have at least 50% obstruction in at least one coronary artery, have a clinical history of unstable angina, previous acute coronary syndromes with or without persistent ST-segment elevation in addition to the presence of several risk factors for cardiovascular disease.

The following data were also obtained: age, sex, and the presence of risk factors (cigarette, smoking, hypertension (diagnosed according to the JNC criteria (Joint National Committee on Prevention, Detection, Evaluation, and Treatment of High Blood Pressure)), diabetes mellitus (diagnosed and classified according to American Diabetes Association criteria (Expert Committee on the Diagnosis and Classification of Diabetes Mellitus, 1997)) and dyslipidemia (defined as low-density lipoprotein-cholesterol (LDL-C) > 2.6 mmol/L, low high-density lipoprotein-cholesterol (low HDL-C) (<1.03 mmol/L in men and <1.29 mmol/L in women) and/or triglycerides >1.7 mmol/L). Hypercholesterolemia was defined as a cholesterol level ≥ 5.68 mmol/L, a triglyceride concentration ≥ 2.28 mmol/L or when receiving a lipid lowering drug. Weight and height were measured using a standard scale and waist to hip ratio (WHR) was calculated. Body mass index (BMI) was calculated from the equation: BMI = weight/height2 (kg/m2). Major requirements for enrolment in all patients were: absence of infectious or acute/ chronic inflammatory diseases, known malignancy, absence of acute/chronic renal failure, or hepatic failure, percutaneous coronary intervention and cerebrovascular accident. All controls were recruited at several diabetes screening days. Among these people none had any cardiovascular risk factors (no hypertension, no diabetes, no obesity, no hyperlipidemia, no family history except smoking and menopause with a reduced numbers and no medication use). Thereafter, blood samples were collected from all these fasting people and analysis of biochemical parameters, as mentioned in our paper, was made. None of the subjects and controls was using an antioxidant therapy, vitamin supplementation or hormonal replacement therapy for the post menopausal women. The Institution Ethics Committee for studies on human subjects approved this study, and informed consent was obtained from all patients and controls before their enrolment.

### Blood sample preparation

Fasting blood samples were collected from all participants and all analysis were assayed in plasma. Blood samples were collected into EDTA tube then centrifuged at 3000 rpm for 10 min at 4 °C. Plasma was collected and stored at −80 °C until biochemical analysis.

Plasma glucose concentration was evaluated using an enzymatic kit (glucose oxidase, Randox, Antrim, UK), glycosylated hemoglobin (HbA1c) by an exchange microcolumn chromatographic procedure (Biosystems, Barcelona, Spain), lipid parameters were determined as described previously by Smaoui et al. [12] and high-sensitivity C Reactive Protein (hs-CRP) levels was determined using an immunonephelometric method (Behring N Latex C Reactive Protein Mono-Analyzer; Behring Diagnostic, Marburg,Germany). In fact polystyrene particles coated with monoclonal antibodies specific to human CRP are aggregated when mixed with samples containing CRP. These aggregates scatter a beam of light passed through the sample. The intensity of the scattered light is proportional to the concentration of the relevant protein in the sample. The results were evaluated by comparison with a standard of a known concentration.

### Coronary angiographic data and Gensini score

All coronary angiography results were interpreted for the presence, extent and severity of CAD by two experienced interventional cardiologists. Patients were classified into CAD and non CAD patients according to angiographic results (≥ 50% obstruction in ≥1 coronary artery). GS was used to assess the severity of CAD: it grades narrowing of the lumen of the coronary artery and scores it as 1 for 1%–25% narrowing, 2 for 26%–50% narrowing, 4 for 51%–75%, 8 for 76%–90%, 16 for 91%–99% and 32 for a completely occluded artery. This score was then multiplied by a factor according to the importance of the coronary artery. The multiplication factor for a left main stem (LMS) lesion was 5. It is 2.5 for proximal left anterior descending artery (LAD) and proximal circumflex artery (CX) lesions, 1.5 for a mid-LAD lesion, and 1 for distal LAD, mid/distal CX and right coronary artery lesions. The multiplication factor for any other branch is 0.5. The points were then added and the total Gensini score of each patient was calculated. The severity of disease was expressed as the sum of the scores for the individual lesions [1].

### Fatty acid profile analysis

Plasma FA were analyzed as fatty acid methyl esters (FAMEs) by gas chromatography analysis. FA were extracted from plasma as previously described by Folch et al. with slight modifications [13]. Individual FAMEs were separated and quantified by gas chromatography using a Model 5890 Series II instrument (Hewlett-Packard, Palo Alto, CA, USA) equipped with a flame ionization detector and a fused silica capillary column SP-2560 (100 m length, 0.25 mm i.d., and 0.2-μm film thickness) (with heptadecanoic acid, 17:0, used as the internal standard). 1 μL of each sample was injected into the gas chromatography system.

The initial oven temperature was programmed to 140 °C for 5 min. Then, it increase to 240 °C at a rate of 4 °C / min. The injector and detector temperatures were 260 °C.

FAMEs were identified by comparing their retention times with those of individual standards used in mixture (Supelco 37 Component FAME Mix, Palo Alto, USA). The FA composition was reported as a relative percentage of the total peak area using a HP Chemstation integrator.

### Dietary survey

Food intakes were estimated by two dietitians using an open-ended, interview-administered diet history. Subjects were asked on their daily diet over a week period. They were also asked on amounts, frequencies and variations in consumption. Energy and nutrient intakes were calculated by using the software Nutritionist IV Computer Analysis Program [14].

### Statistical analysis

All statistical analyses were performed using the Statistical Package for Social Sciences SPSS 18.0 for Windows (SPSS Inc., Chicago, IL, USA). Data were presented as frequencies and percentages for categorical variables, mean ± standard deviation (SD) for continuous variables, or median (interquartile range) for several data that were not normally distributed; numeration data were presented as constituent ratio. All continuous variables were tested for normal distribution and homogeneity of variance. Non parametric Mann-Whitney test was used. Differences between two groups were assessed using the chi-square and unpaired t-tests. Correlation between continuous variables was determined by Pearson correlation coefficients. The Spearman correlation test was also used to evaluate the relationships between various parameters. Multivariate logistic regression model was used to assess the relationship between Gensini score and different risk factors as well as different fatty acids. *p* values <0.05 and <0.001 were considered as statistically significant.

## Results

### Characteristics of study subjects

Among the 270 participants, 111 patients had angiographically-proven CAD (CAD group), 39 had non-obstructive or no coronary atherosclerotic lesions (non-CAD group) and 120 were control subjects. Clinical and biochemical characteristics of the three groups were shown in Table 1.

**Table 1:** The demographic, clinical and biochemical characteristics of controls and patients

Variables	Controls (*n* = 120)	Non-CAD group (*n* = 39)	CAD group (*n* = 111)
Sexe n (%)			
Male	53 (44)	16 (41)	80 (72)**
Femelle	67 (56)	23 (59)**	31 (28)**
Age (years)	57.10 ± 12.65	60.20 ± 9	60.83 ± 9
Current smoking n (%)	14 (12)	7 (18)	38 (34)**
Diabetes n (%)	0 (0)	15 (38)**	43 (39)**
Hight blood pressure n (%)	0 (0)	25 (64)**	52 (47)**
Hyperlipidemia n (%)	0 (0)	15 (38)**	50 (45)**
BMI (kg/m^2^)	27.73 ± 3.43	30.23 ± 4.65*	29.44 ± 4.28*
Obesity n (%)	0 (0)	22 (56)**	46 (41)**
Menopause n (%)	26 (22)	20 (51)**	28 (25)
family history n (%)	0 (0)	10 (26)**	24 (22)**
Medications n (%)			
Statins	0 (0)	16 (41)**	35 (31)**
antiagreguant	0 (0)	20 (51)**	66 (59)**
ACE-I	0 (0)	21 (54)**	65 (59)**
BETA-Bloker	0 (0)	20 (51)**	56 (50)**
Hypoglycemic drug	0 (0)	7 (18)**	31 (30)**
Fibrate	0 (0)	8 (20)**	36 (32)**
Gensini score		0 (0, 3)	28 (13, 52)
Total cholesterol (mmol/L)	4.80 ± 0.78	4.95 ± 0.86	4.97 ± 0.99
LDL-C (mmol/L)	3.1 ± 0.81	3.13 ± 0.85	3.11 ± 0.92
HDL-C (mmol/L)	1.22 ± 0.44	1 ± 0.22*	1.01 ± 0.24**
Triglycerides (mmol/L)	1.33 ± 0.38	1.8 ± 1.03*	1.83 ± 0.86**
Creatinine (μmol/L)	85.14 ± 14.06	84.81 ± 22.68	100.16 ± 23.45**
Urea (μmol/L)	5.31 ± 1.68	5.9 ± 2.21	6.62 ± 3**
hs-CRP (mg/L)	1.22 ± 0.95	2.33 ± 1.5**	3.03 ± 2.26**
Glucose (mmol/ L)	5.1 ± 0.8	8.14 ± 5.43**	9.62 ± 4.7**
Hemoglobin A1c (%)	5.24 ± 0.92	7.46 ± 3.01**	8.61 ± 3.06**
ApoA1 (g/l)	1.30 ± 0.15	1.13 ± 0.16**	1.12 ± 0.17**
APoB (g/l)	0.6 ± 0.14	0.75 ± 0.14**	0.77 ± 0.24**
Total cholesterol /HDL	4.31 ± 1.45	5.21 ± 1.61*	5.19 ± 1.76**
Triglycerides /HDL	1.23 ± 0.57	2.04 ± 1.85*	1.96 ± 1.09**
LDL/HDL	2.87 ± 1.30	3.27 ± 1.18*	3.3 ± 1.48*
ApoB/ApoA1	0.47 ± 0.13	0.68 ± 0.19**	0.7 ± 0.21**

Data are expressed as n (%) (number of persons, (percent)), mean ± SD (standard deviation) or median (interquartile, range). A significant difference between controls and coronary artery disease patients is indicated by * (Mann Whitney Test). *: *p* < 0.05. A significant difference between controls and patients (Non-CAD group and CAD group) is indicated by * (*p* < 0.05) or ** (*p* < 0.001). ApoA1: Apolipoprotein A1; ApoB: Apolipoprotein B; CAD: coronary artery disease; BMI: body mass index; LDL-C: low-density lipoprotein-cholesterol; HDL-C: high-density lipoprotein-cholesterol; hs-CRP: high sensitive-C reactive protein; ACE-I: angiotensin-converting enzyme inhibitor

The mean age of control group was 57.10 ± 12.65 years old versus 60 ± 9 years old for the two groups of patients, and the only risk factor for coronary artery disease in this group was smoking with a percentage of 12%.

In our study, a marked male predominance was noticed in CAD group with 80 men among 111 (72%). High blood pressure was the most dominant risk factor of CHD in the two groups of patients followed by hyperlipidemia, obesity and diabetes. The GS ranged from 0 to 155 with a median of 28 (13, 52) for the CAD group and 0 (0, 3) for non-CAD group.

In the CAD patients, TG level was significantly increased and HDL-C level was decreased significantly as compared with the controls (*p* < 0.001). However, no significant differences in LDL-C and TC levels were observed between the two groups.

An increase of total cholesterol (TC) /HDL-C, TG/ HDL-C and LDL-C/HDL-C ratio was observed in the two groups of patients comparatively to controls. In addition, a statistically significant decrease of apolipoprotein A1 (ApoA1) and increase of apolipoprotein B (ApoB) levels were found in the two groups of patients compared to controls (*p* < 0.001). The ratio was also significantly increased in the two groups compared to the controls (CAD group: 0.7 ± 0.21; non-CAD group: 0.68 ± 0.19; controls: 0.47 ± 0.13, *p* < 0.001).

In the two groups of patients, HbA1c, hs-CRP and serum creatinine levels were significantly increased when compared to the controls (*p* < 0.001).

In addition, most of the patients in the CAD group and non-CAD group received antiagregant medication (59% vs 51%), angiotensin-converting enzyme inhibitor (ACE-I) (59% vs 54%), Beta-Bloquers (50% vs 51%), fibrate (32% vs 20%) and statins medication (31% vs 41%).

### Correlation and logistic regression between Gensini score, lipid profile, and glycemic parameters in controls CAD and non-CAD groups

Spearman’s correlation coefficient was used to evaluate the correlation between the **GS** and several CAD risk factors. There was a significant negative association between HDL-C levels and the GS for the two groups of patients (non- CAD: *r* = −0.3, *p* < 0.05; CAD group: *r* = −0.324, *p* < 0.05) and a significant positive association between this score and the glucose level in the two groups of patients (non- CAD: *r* = 0.318, *p* < 0.05; CAD group: *r* = 0.389, *p* < 0.05) (Table 2).

**Table 2:** Correlation between Gensini Score, lipid profile, and glycemic parameters in CAD and non-CAD groups

	non-CAD group		CAD group	
	r	*p-*value	r	*p*-value
Glucose (mmol/L)	0.318	**0.048**	0.389	**0.023**
Hemoglobin A1c (%)	0.305	0.05	0.048	0.619
Total cholesterol (mmol/L)	0.02	0.906	0.017	0.858
LDL-C (mmol/L)	−0.016	0.925	0.005	0.955
HDL-C (mmol/L)	−0.31	**0.049**	−0.324	**0.04**
Triglycerides (mmol/L)	0.22	0.178	−0.069	0.474
ApoA1 g/L	0.091	0.583	0.017	0.862
ApoB g/L	−0.254	0.119	0.036	0.708

r: Spearman’s correlation coefficients

Therefore, all of these variables were entered in the multiple logistic regression model. Those, which were statistically different, can be seen in Table 3.The multivariable odds ratios ranged from 0.987 for glucose to 1.481 for HDL-C. All multivariable coefficients were statistically significant (*p* < 0.05). Glucose, Hemoglobin A1c, LDL-C and HDL-C were independent risk factors of vascular severity for CAD patient.

**Table 3:** Multivariate logistic analysis of the risk factors associated with the vascular severity

Risk factors	OR^1^	Confidence interval (95%CI)
Glucose (mmol/L)	0.987*	0.847–1.151
Hemoglobin A1c (%)	1.183*	0.924–1.513
LDL-C (mmol/L)	1.011*	0.674–1.700
HDL-C (mmol/L)	1.481*	0.332–6.602

^1^OR = Odds ratio

*p < 0.05

### Evaluation of dierty intake in coronary patients and controls

A balanced diet plays an important role and contributes effectively to improve lipid balance. To study the effect of diet on various clinical and biological parameters of coronary, a dietary survey was conducted in coronary patients and controls. The results of the daily intake of various nutrients in both patients and controls showed a statistically significant difference in daily calorie intake between control, CAD and non-CAD. This is associated with a slight decrease of carbohydrate intake which remains no significant in both patient groups compared to controls and an important significant variation in the simple carbohydrates intake which decreased significantly and very significantly in non-CAD and CAD respectively compared to control (non-CAD: 27.99 ± 6.64, CAD: 28.34 ± 6.53 and controls: 35.66 ± 5.77). Knowing that carbohydrate intake covers about 55–57% of total energy intake, which is a characteristic of a Mediterranean diet **(**Table 4 **)**. The difference also lies in MUFA consumption. However, no significant changes were observed in PUFA, SFA and cholesterol intakes, which does not exceed the recommended intake (300 mg / d), in both CAD and non-CAD groups compared to controls. Fat intake covers about 30% of total calories intake which are about 10% of MUFA, 6% of PUFA and 10% of AGS with a highly significant of the ration MUFA / SFA in CAD and non-CAD compared to controls. Protein intake which is of the order of 15% has also shows no significant variation between patients and controls **(**Table 4 **)**. The dietary of the two groups of patients (CAD and non-CAD) is also characterized by a significant increase in antioxidant vitamin E compared to the control group (non-CAD: 10.23 ± 1.74, CAD 9.98 ± 1,51 and controls: 8.09 ± 2.12 mg) despite that its contribution is still below the recommended intake of 15 mg/d. For other micronutrients, such as vitamins B1 and C, calcium, phosphorous, magnesium, potassium and zinc, no significant variation was observed between the two groups of patients and controls. For dietary fiber, although no change was observed, the contribution remains lower than the recommended intake which is about 25 g/d **(**Table 4 **)**.

**Table 4:** Nutrient intakes of controls, non-CAD and CAD groups

Nutrient	Controls (*n* = 120)	non-CAD group (*n* = 39)	CAD group (*n* = 111)
kcalories/days	**2172.52 ± 249.6**	2048.59 ± 326.98*	2028.96 ± 284.71*
Protein			
g	53.39 ± 7.3	51.49 ± 12.04	52.24 ± 11.59
%	15.3 ± 2.26	14.54 ± 2.81	14.87 ± 2.89
Carbohydrate			
g	202.36 ± 33.05	199.37 ± 32.8	197.38 ± 7.3
%	57.29 ± 4.58	56.32 ± 3.99	55.6 ± 4.5
Simple carbohydrate			
g	**35.66 ± 5.77**	27.99 ± 6.64*	28.34 ± 6.53**
%	**7.73 ± 1.61**	7.3 ± 2.08*	7.12 ± 1.82*
Lipid			
g	**95.48 ± 7.94**	102 ± 7.5*	**100.91 ± 6.51**
%	**27.41 ± 3.29**	29.13 ± 3.24*	29.13 ± 3.7**
SFA (%)	9.54 ± 1.12	9.93 ± 1.49	9.78 ± 1.36
MUFA (%)	**18.09 ± 2.12**	20.43 ± 1.74*	19.98 ± 1.36*
PUFA	5.17 ± 0.61	5.78 ± 0.5	5.71 ± 0.43
MUFA/SFA	**1.90 ± 0.11**	2.07 ± 0.31**	2.08 ± 0.35**
Cholesterol (mg)	211.28 ± 28.67	212.65 ± 22.87	213.42 ± 28.4
Fiber (g)	20.74 ± 3.07	20.08 ± 2.47	20.43 ± 3.12
Folates (μg)	152.54 ± 16.42	159.4 ± 15	159.3 ± 16.5
Vitamin B1 (mg)	0.51 ± 0.06	0.52 ± 0.04	0.52 ± 0.04
Vitamin C (mg)	87.89 ± 10.31	90.38 ± 8.04	89.11 ± 7.6
Vitamin E (mg)	**8.09 ± 2.12**	10.23 ± 1.74**	9.98 ± 1.51**
calcium (mg)	517.03 ± 60.68	528.07 ± 41.5	521.4 ± 46.67
Potassium (mg)	2585.13 ± 303.39	2590.37 ± 207.51	2557 ± 233.36
Phosphorus (mg)	952.86 ± 109.47	960.45 ± 137.93	945.71 ± 129.66
Magnesium (mg)	253.44 ± 29.74	253.37 ± 20.34	250.1 ± 22.88
Zinc (mg)	9.53 ± 1.09	9.93 ± 1.35	9.78 ± 1.28

SFA: Saturated fatty acids, MUFA: Monounsaturated fatty acids, PUFA: Polyunsaturated fatty acids, *p < 0.05

### Evaluation of the relationship between dietary intakes and lipid parameters in CAD and non-CAD patients

In the group of non-CAD, a statistically significant positive correlation was observed between daily intake of simple carbohydrates and plasma cholesterol levels with a Spearman coefficient *r* = 0.328 and *p* = 0.042. However, a negative correlation between the daily fat intake and plasma cholesterol levels (*r* = −0.364; *p* = 0.023) was observed. For the group of CAD, the correlation was negative between daily intake of SFA and HDL-C (*r* = −0.191 and *p* = 0.044). In addition, the daily lipid consumption is positively correlated with the plasma levels of HDL-C with a Spearman coefficient *r* = 0.189 and *p* = 0.047 **(**Table 5 **)**. However, all of these variables were entered in the multiple logistic regression model and no statistical differences were observed in all parameters.

**Table 5:** Spearman correlation between glycemic and lipid parameters and nutrient intake of both CAD and non-CAD groups

	non-CAD group (*n* = 39)							CAD group (*n* = 111)						
	Protein (%)	Carbohydrate (%)	Simple carbohydrate (%)	Lipid (%)	SFA (% cal)	MUFA (% cal)	PUFA (% cal)	Protein (%)	Carbohydrate (%)	Simple carbohydrate (%)	Lipid (%)	SFA (% cal)	MUFA (% cal)	PUFA(% cal)
Hemoglobin A1c (HbA1c, (%))	−0.114	0.030	−0.258	0.054	−0.263	−0.213	−0.213	−0.171	0.132	0.131	−0.085	0.080	0.132	0.119
Glucose (mmol/L)	−0.029	0.072	−0.161	−0.018	−0.160	−0.184	−0.184	−0.149	0.080	0.086	−0.069	0.032	0.100	0.110
Triglycérides (mmol/L)	−0.198	0.156	0.014	−0.044	0.018	0.044	0.044	−0.092	−0.033	−0.143	0.058	−0.137	0.028	−0.033
HDL-C (mmol/L)	0.185	0.019	0.204	−0.107	0.206	−0.092	−0.092	−0.101	−0.075	−0.154	0.189*	−0.191*	−0.019	−0.038
LDL-C (mmol/L)	0.202	−0.035	0.210	−0.233	0.196	−0.100	−0.100	−0.012	0.041	0.058	−0.054	0.057	0.078	0.017
Cholestérol total (mmol/L)	0.158	0.118	0.328*	−0.364*	0.299	−0.062	−0.062	−0.070	−0.024	−0.076	0.054	0.001	0.019	−0.052

SFA: Saturated fatty acids, MUFA: Monounsaturated fatty acids and PUFA: Polyunsaturated fatty acids. *p < 0.05

### The plasmatic fatty acid profile evaluation by gas chromatography

To establish a profile of FAs, analyses by gas chromatography were conducted on the plasma of non-CAD, CAD patients and controls.

The FA profile in the plasma is given in Table 6. Analysis of plasma FA composition in patients showed a significant difference of total SFA, 13:0, 16:0, and 24:0 in the two groups of patients compared to controls (*p* < 0.001). However a decrease of 14:0, 23:0 and 26:0 was revealed in patients compared to control subjects.

**Table 6:** Plasma fatty acid composition and desaturase activity index of controls, non-CAD and CAD patients

	Controls (*n* = 120)	non-CAD patients (*n* = 39)	CAD patients (*n* = 111)
Total SFA	34.01 ± 5.27	37.23 ± 3.72**	37.14 ± 3.41**
lauric acid, 12:0	0.15 ± 0.12	0.24 ± 0.38	0.45 ± 0.80**
Tridecylic acid, 13:0	0.16 ± 0.25	0.33 ± 0.5*	0.36 ± 0.63**
myristic acid, 14:0	4.17 ± 1.39	3.43 ± 1.43*	3.64 ± 1.37*
pentadecylic acid, 15:0	0.3 ± 0.18	0.38 ± 0.18*	0.4 ± 0.25**
Palmitic acid, 16:0	15.65 ± 5.35	20.21 ± 3.02**	19.63 ± 3.39**
Stearic acid, 18:0	8.11 ± 1.47	8.26 ± 1.39	7.83 ± 1.17
Arachidic acid, 20:0	0.26 ± 0.20	0.29 ± 0.2	0.38 ± 0.32**
Behenic acid, 22:0	0.41 ± 0.28	0.30 ± 0.26*	0.44 ± 0.35
Tricosylic acid, 23:0	1.12 ± 0.58	0.44 ± 0.26*	0.91 ± 0.37*
Lignoceric acid, 24:0	0.25 ± 0.2	0.44 ± 0.26**	0.47 ± 0.35**
Cerotic acid, 26:0	3.4 ± 1.05	2.45 ± 0.97**	2.58 ± 1.07**
Total MUFA	20.25 ± 3.48	25.32 ± 3.57**	24.55 ± 3.28**
Myristoleic acid, 14:1n-5	0.97 ± 0.39	1.35 ± 0.39**	1.42 ± 0.57**
Palmitoleic acid, 16:1 n-5	1.33 ± 0.32	2.82 ± 1.1**	2.67 ± 0.88**
Oleic acid, 18:1n-9	13.18 ± 3.71	16.64 ± 3.37**	15.95 ± 3.28**
cis-Vaccenic acid, 18:1 n-7	2.21 ± 0.73	2.08 ± 0.48	2.08 ± 0.57
Eicosenoic acid, 20:1 n-9	0.8 ± 0.46	0.64 ± 0.26*	0.7 ± 0.37*
Erucic acid, 22:1n-9	0.38 ± 0.25	0.3 ± 0.32*	0.32 ± 0.21*
Nervonic acid, 24:1 n-9	1 ± 0.81	0.93 ± 0.97	0.86 ± 1.04*
Total PUFA	43.05 ± 5.9	33.92 ± 4.47**	34.12 ± 2.93**
Total PUFA n-3	4.47 ± 1.3	5.06 ± 1.16*	4.74 ± 1.44
Alpha-linolenic acid, 18:3n-3	0.47 ± 0.37	0.65 ± 0.51*	0.7 ± 0.54*
Eicosatrienoic acid, 20:3n-3	2.37 ± 0.71	2.48 ± 0.92	2.23 ± 1
Eicosapentaenoic acid, 20:5n-3	0.84 ± 0.77	1.06 ± 0.88*	0.96 ± 0.72*
Docosahexaenoic acid, 22:6n-3	0.78 ± 0.39	0.86 ± 0.44	0.84 ± 0.36
Total PUFA n-6	38.58 ± 6.26	28.86 ± 4.62**	29.38 ± 3.15**
Linoleic acid, 18:2n-6	31.89 ± 6.44	20.20 ± 4.78**	20.56 ± 3.371**
c-linolenic acid, 18:3n-6	0.65 ± 0.34	0.87 ± 0.35**	0.96 ± 0.42**
Dihomo-c-linolenic acid, 20:3n-6	0.49 ± 0.25	0.52 ± 0.2	0.57 ± 0.29
Arachidonic acid, 20:4n-6	5.55 ± 1.59	7.25 ± 2.02**	7.28 ± 1.77**
Total UFA	63.30 ± 5.24	59.25 ± 3.67**	58.67 ± 3.60**
Total PUFA/Total SFA	1.32 ± 0.37	0.92 ± 0.18**	0.93 ± 0.13**
Desaturation index			
20:4n-6/20:3n-6 (D5D)	13.28 ± 8.14	15.72 ± 7.47*	14.56 ± 6.8*
20:4n-6/18:2n-6 (D6D)	0.18 ± 0.07	0.47 ± 0.43**	0.37 ± 0.13**
18:3n-6/18:2n-6 (D6D)	0.02 ± 0.01	0.05 ± 0.07**	0.05 ± 0.02**
16:1/16:0 (D9D-16)	0.09 ± 0.04	0.14 ± 0.05**	0.14 ± 0.05**
18:1n-9/18:0 (D9D-18)	1.70 ± 0.66	2.07 ± 0.57**	2.08 ± 0.53**
14:n-5/14:0	0.27 ± 0.16	0.62 ± 0.16**	0.42 ± 0.17**
SFA/UFA	0.54 ± 0.13	0.63 ± 0.10**	0.64 ± 0.17**

Data are expressed as relative values (%): mean % of total Plasma fatty acid ± SD. A Mann Whitney test was used. Significant difference between controls and patients is indicated by *: *p* < 0.05 and **: *p* < 0.001. SFA: saturated fatty acid; MUFA: monounsaturated fatty acid; PUFA: polyunsaturated fatty acid; PUFA n-3: polyunsaturated n-3 fatty acid; PUFA n-6: polyunsaturated n-6 fatty acid; UFA: unsaturated fatty acids; C16:1/C16:0 and C18:1/C18:0: ∆9 desaturation index; C20:4/C18:2: ∆6 desaturation index; C20:4/C20:3: ∆5 desaturation index

Total MUFA was higher in the two groups of patients than controls. It was associated with a significant increase of 14:1n-5, 16:1n-5 and 18:1n-9 (Oleic acid: the most abundant fatty acid). However, a significant decrease of 20:1n-9, 22:1n-9 and a slight decrease of 24:1n-9 were observed in patients relatively to controls.

Total PUFA were decreased in the two groups of patients, mainly due to the significant decrease of total n-6 PUFA. This reduction results especially from the significant decrease of linoleic acid, (LA, 18:2n-6) precursor of n-6 PUFA (non-CAD group: 20.20 ± 4.78; CAD group: 20.56 ± 3.371; controls: 31.89 ± 6.44 (*p* < 0.001)). However, a significant increase in the level of arachidonic acid, (AA, 20:4n-6) and c-linolenic acid (CLA, 18:3n-6) in the two groups of patients was observed compared to the controls, but the proportion was still very low compared to LA.

For the n-3 PUFA, a significant increase of alpha-linolenic acid (ALA, 18:3 n-3) and EPA was observed, but the proportion of 20:3 n-3 and DHA remained unchanged.

As a consequence, D9D activities, estimated by the ratio 16:1/16:0 and 18:1n-9/18:0 and D5D activity, estimated by the ratio 20:4n-6/20:3n-6, were significantly higher in the two groups of patients (*p* < 0.001). A significant increase of D6D activity, presented as 20:4n-6/18:2n-6 and 18:3n-6/18:2n-6 was observed in the two groups of patients (*p* < 0.001).

Significant increase of cell membrane viscosity- index (SFA/UFA) was revealed in the two groups of patients comparatively to controls (*p* < 0.001).

### Relationship between plasma fatty acids and the severity of CAD evaluated by Gensini score

Significant negative correlation was revealed between the severity of CAD, estimated by GS and several plasma fatty acids, especially; Total MUFA, n-3 PUFA, 22:6n-3, 18:3n-6, 20:3n-6 and total PUFA (Table 7). However, a positive correlation with 18:0 and 22:1n-9 was found between these FA and GS.

**Table 7:** Spearman correlation between plasma fatty acids and the severity of CAD evaluated by Gensini score

	r*	*p*-value
**Total SFA**	−0.152	0.112
Palmitic acid, 16:0	−0.104	0.277
Stearic acid, 18:0	**0.211**	**0.026**
**Total MUFA**	**−0.185**	**0.045**
Palmitoleic acid, 16:1	−0.101	0.290
Oleic acid, 18:1n-9	−0.078	0.414
Erucic acid, 22:1n-9	**0.231**	**0.015**
**Total PUFA n-3**	**−0.250**	**0.008**
a -linolenic acid, 18:3n-3	−0.001	0.992
Eicosapentaenoic acid, 20:5n-3	−0.133	0.163
Docosahexaenoic acid, 22:6n-3	**−0.228**	**0.016**
**Total PUFA n-6**	−0.136	0.155
Linoleic acid, 18:2n-6	−0.111	0.244
c-linolenic acid, 18:3n-6	**−0.235**	**0.013**
Dihomo-c-linolenic acid, 20:3n-6	**−0.215**	**0.023**
Arachidonic acid, 20:4n-6	−0.141	0.140
**Total PUFA**	**−0.185**	**0.045**
**Total PUFA/Total SFA**	−0.077	0.419
**Desaturation index**		
20:4n-6/20:3n-6 (D5D)	0.134	0.162
20:4n-6/18:2n-6 (D6D)	−0.021	0.830
18:3n-6/18:2n-6 (D6D)	−0.159	0.095
16:1/16:0 (D9D-16)	0.017	0.857
18:1n-9/18:0(D9D-18)	0.125	0.192
14:n-5/14:0	−0.096	0.318
**SFA/UFA**	−0.005	0.958

*: r = Spearmen’s correlation coefcients

Mulivariate logistic analysis schowed that total MUFA, 18:2n-6, 20:4n-6 and 22:6n-3 were independent risk factors of vascular severity for CAD patient. The multivariable odds ratios ranged from 0.972 for 20:4n-6 to 1.516 for 22:6n-3. All multivariable coefficients were statistically significant (*p* < 0.05) (Table 8).

**Table 8:** Multivariate logistic analysis of plasma fatty acids associated with the vascular severity evaluated by Gensini score

Fatty acids	OR^a^	Confidence interval (95%CI)
Total MUFA	1.155*	0.867–1.538
Linoleic acid, 18:2n-6	1.38*	1.03–1.847
Arachidonic acid, 20:4n-6	0.972*	0.491–1.212
Docosahexaenoic acid, 22:6n-3	1.516*	1.053–9.658

^a^OR = Odds ratio

*p < 0.05

## Discussion

Atherosclerosis is one of the major events leading to CAD development. Among the various factors leading to atherosclerosis, major risk factors are high cholesterol level, hypertension, metabolic syndrome and diabetes mellitus [15]. Dyslipidemia is a major risk factor for CAD. It is strongly associated with the incidence, development of atherosclerosis and vascular severity [16]. Alterations of lipid profile, disturbed FAs metabolism and inflammation can promote lipotoxicity.

In order to evaluate the lipid related risk factors for CAD development, major guidelines have proposed the diagnostic utility of LDL-C, TG, and lipid ratios such as TC/HDL-C, TG/ HDL-C and LDL-C/HDL-C [17]. In our study, the analysis of conventional lipid parameters showed that in the CAD group, plasma TG levels were significantly increased and HDL-C levels were significantly decreased as compared with the controls. Atherogenic lipid ratios (TC/HDL-C, TG/ HDL-C and LDL-C/HDL-C) were increased in the two groups of patients comparatively to controls. Those observations are confirmed by previous studies [18, 19].

Accordingly, analysis of specialized lipid parameters showed statistically significant increase in the ApoB/ ApoA1 ratio which associated with the development of clinical cardiovascular disease and with early atherosclerosis onset [20]; moreover is considered the stronger predictor of vascular events [21].

All these results show that there is a clear alteration of the lipid profile in CAD patients, and a close association of apolipoprotein metabolism with the development and progression of atherosclerosis.

Analysis of the severity of CAD assessed using the Gensini score (GS) showed, a significant positive association with mean glucose level. This was in accordance with different studies showing that diabetes is correlated with the GS [22] and might induce coronary artery endothelium dysfunction and then contribute to the development of CAD [23].

Furthermore, significant negative association with mean serum HDL-C level in the two groups of patients (non- CAD and CAD group) shows that GS is appropriate and well adapted to prove a metabolic disorder associated with cholesterol metabolism and carbohydrate metabolism in CAD patient.

The study of FA composition and the estimation of desaturases activities could provide new information about the altered FA metabolisms. The plasma FA levels and GS were higher in CAD patients than in non-CAD patients and controls.

A significant increase of the SFA and MUFA in the CAD and non-CAD groups compared to controls was found. According to experimental studies [24] and clinical trials [25] strong association has been reported between cardiovascular disease and SFAs. Despite that no variation was observed in the daily SFA intake between patients and controls a negative correlation between this SFA intake and HDL-C was observed in the CAD group. Varied mechanisms have been proposed to account for pro-inflammatory effect of dietary SFA. In addition, as we found, Blankenhorn et al. [26] reported an association between CAD progression and intake of lauric, oleic, and linoleic acids.

Mente et al. [27] showed a significant inverse correlation between MUFA intake and CAD events, this can be the result of the effect of MUFA as a mediator for the beneficial effects of reduced SFA intake. The stearic acid level was unchanged in patients comparatively to controls and this may be due to diet intake followed by patients.

According to our study of the estimated of dietary behavior intake of various nutrients of patients and controls, no significant change was observed at intakes of SFA and cholesterol, which does not exceed the recommended intake (300 mg / d), in both CAD and non-CAD groups compared to controls. In addition, fat intake covers about 30% of total energy intake which are about 10% of MUFA, 6% of PUFA and 10% of SFA with a highly significant ratio of MUFA / SFA in CAD and non-CAD groups compared to controls. Protein intake which is in the order of 15% didn’t show any significant variation between patients and controls. While carbohydrate intake covers about 55–57% of total energy intake, which is characteristic of a Mediterranean diet. The alimentation of two groups of patients (CAD and non-CAD) is also characterized by a significant increase in antioxidant vitamin E compared to the control group. These results highlight the Mediterranean diet as the food consumption patterns of the Tunisian population which is generally defined as rich in fruits, vegetables, and cereals, with relatively low intakes of meat, and olive oil as the main source of added fat. It has been suggested that this diet reduce coronary events by decreasing stearic acid intake [28].

This diet is characterized by a high intake of carbohydrates covering 51 to 56% of total calorie intake and a relatively low consumption of fat (30 to 37%) of total energy intake with only 10% are saturated AG. This restriction of saturated fat has the advantage of reducing dietary intake of cholesterol below 300 mg / day as usually provided by the same food. This diet is also rich in MUFA (> 50% of fat intake) contained mainly in olive oil, an essential component of the Mediterranean diet [29].

In addition, a significant decrease of total PUFA, PUFA n-6 and especially linoleic acid (LA) was observed in the two groups of patients. Different studies proved that higher intake of PUFA n-6 reduce the risk of CHD [30, 31] and showed that LA which is the predominant dietary n-6 PUFA have blood cholesterol benefits [25], and that higher dietary PUFA (predominantly LA) is associated with lower CAD risk in prospective cohort studies [5].

Furthermore, in the same class of PUFA n-6, we observed, a significant increase of γ -linolenic acid (GLA), arachidonic acid (AA) and a slight increase of dihomo-γ-linoleinc acid (DGLA) in CAD and non-CAD patients (*p < 0.001*) compared to controls. This increase may be one of the reasons for the development of atherosclerotic plaques. This may be due to the important role played by AA as a direct precursor of strong inflammatory eicosanoids. However the impact of n-6 PUFA on health especially CAD, including GLA, DGLA, and AA, remains poorly established.

The study of Sayantani et al. was the only study showing that dietary GLA feeding resulted in significant decrease in serum TG and very low density lipoprotein (VLDL) cholesterol in addition to the significant increase in HDL-C [32].

In addition, Horrobin has pointed out that low DGLA levels may be strong markers of CAD risk [33]. Furthermore, some studies underlined that increased serum levels of n-3 PUFAs, associated with a decreased incidence of cardiovascular events and mortality in older patients [34].

Moreover, as expected, negative correlations were found between different FA (total MUFA, PUFA n-3, Total PUFA, GLA, DGLA and DHA) and GS. In addition, the other important observation was the positive association of GS with stearic and erucic acid which was known to cause cardiac lipidosis [32]. Accordingly, our results leading us to state that the GS can be also appropriate to evaluate the metabolic disorder related to FA.

The PUFA profile in plasma and erythrocytes does not only reflect dietary intake, but is also strongly dependent the endogenous metabolism of PUFAs. In fact, an increase in the index of cell membrane viscosity (SFA/UFA) was found in our study. Changes in membrane viscosity, are known to be associated with the alteration in physiological process, and has been associated with disease states. They are also known as good indicators of cell viability [35]. Furthermore, membrane viscosity increases have been associated with the development of atherosclerosis, diabetes and hypercholesterolemia [36].

Moreover, the decrease of total UFA level in non CAD and CAD group seem to be associated with the alteration in membrane fluidity, because these changes have been suggested to decrease the membrane fluidity [37].

In addition to the alteration of different FA, higher elongation index and modifications of ∆5, ∆6 and ∆9 desaturation indexes were also observed supporting modifications of FA metabolism. In fact, we found that three desaturase activities (D9D, D5D and D6D) were associated with CAD in Tunisian population. The essential FA such as LA (18:2 n-6) and ALA (18:3 n-3), which are important constituents of cell membranes and influence both inflammatory and atherosclerotic processes are metabolized to AA (20:4 n-6) and EPA (20:5 n-3), respectively, by D5D and D6D desaturases [2]. Since most of the products of the AA pathway have pro-inflammatory effects, increased D5D and D6D activity in the n-6 pathway may promote systemic inflammation and increase the risk of atherosclerosis [7]. SCD activities, both D9D-16 and D9D-18, were significantly higher in both groups of patients (with or without CAD) than control subjects, and the main product, 16:0, was also increased in the two groups. Our results are consistent with the study of Si-Wei Li et al. performed on a Chinese Han population [38].

This result was in correlation also with a previous report where an elevation of SCD activity was considered as an independent predictor of cardiovascular risk factors [7]. The Studies of Sampat [39] and Lelliott [40] proposed that there is an association of SCD activity with an increase of lipogenesis and a lipotoxic mechanisms resulting of an ectopic fat deposition and insulin resistance.

A lower level of LA was observed in non-CAD and CAD patients as compared to control group supported the study of Warensjo [9] which showed that the major influencing factor on arterial stiffness was LA. A higher D6D activity (presented by the ratio 20:4 n-6/18:2 n-6), was observed in CAD patients compared to controls (*p* < 0.001). According to the study of Martinelli et al. [41] an increase of this ratio was also observed and was considered as an independent risk factor for CAD confirming our results.

In different cases the modification of FA proportions and alteration of D5D, D6D and D9D activities can result from genetic variants of these enzymes genes. Various studies have shown a big association between plasma PUFAs and FADS gene polymorphisms [42, 43]. The activity of D5D and D6D is also modulated by dietary FA intake, and the level of FAs in the tissues [44]. Furthermore, D9D has also recently been demonstrated as an independent risk factor of cardiovascular disease [9].

## Conclusions

In summary, the alterations of lipid profile in CAD reported in our study showed that individual lipid indexes have a different and important informative value for detecting the presence of CAD. We showed that saturated and unsaturated n-6 and n-3 fatty acids are important determinants of differences in population rates of CHD incidence.

As far as, the analysis of CAD severity, assessed using the GS, a significant positive association with mean glucose level, and a significant negative association with mean serum HDL-C level in the two groups of patients (non- CAD and CAD group) proved that GS is a good indicator to evaluate glycemic and cholesterol disorders associated with HDL-C which is an important factor of CAD and contribute to the vascular severity.

In the relation with some FA and classes of FA, as expected, a negative association was found, between GS and total MUFA, PUFA n-3, total PUFA, GLA, DGLA and DHA in addition to the positive association with stearic and erucic acid.

Our data clearly establish that, GS might be a good indicator to evaluate FA metabolic disorders, glycemic and cholesterol disorders associated with HDL-C.

In our study we proved well that although that Tunisian population appears to follow the Mediterranean diet, CAD patients have an alteration of lipid profile which might be largely due not only to the dietary intake but also to the endogenous metabolism of FAs.

Thus, a comprehensive new strategy to decrease cardiovascular morbidity and mortality should include primarily a cardioprotective diet. The reduction of hyperlipemia and lipid metabolism alteration by appropriate dietary measures is possible. Above all, to this day, no cholesterol-lowering drug has demonstrated to have an effect on cardiovascular morbidity and mortality, without the involvement of dietary measures.

However, the requirement does not limited to this diet, and to the adding lipid-lowering drug but other measures on lifestyle, such as physical activity and smoking cessation which have a favorable effect on dyslipidemia and cardiovascular risk, should be taken.

However, several limitations merit careful considerations. Despite the differences across studies, the association of plasma fatty acid alteration with the severity of CAD lesions has been clearly demonstrated and warrant more clinical studies should be conducted to confirm their clinical applications.

Although our cohort is small, we find significant results; we envisage a prospective study with a larger number of patients. Further research, particularly longitudinal studies, are needed to confirm this hypothesis which opens new perspective for a better understanding of the physiopathogenesis of CAD.

## Abbreviations

AA: Arachidonic acid
ACE-I: Angiotensin-converting enzyme inhibitor
ALA: Alpha-linolenic acid
ApoA1: Apolipoprotein A1
ApoB: Apolipoprotein B
CAD: Coronary artery disease
CLA: C -linolenic acid
D5D: Delta-5-desaturase
D6D: Delta- 6-desaturase
D9D: Delta-9-desaturase
DGLA: Dihomo-γ-linoleinc acid
DHA: Docosahexaenoic acid
EPA: Eicosapentaenoic acid
FA: Fatty acid
FAMEs: Fatty acid methyl esters
GLA: γ -linolenic acid
GS: Gensini score
HbA1c: Glycosylated hemoglobin
HDL-C: High-density lipoprotein-cholesterol
hs-CRP: High-sensitivity C Reactive Protein
LA: Linoleic acid
LDL-C: Low-density lipoprotein-cholesterol
MUFA: Monounsaturated fatty acids;
PUFA: Polyunsaturated fatty acids
SCD: Stearoyl-CoA desaturases
SFA: Saturated fatty acids
